# Predicting the Risk of Re-Offending in Child-to-Parent Violence Using the Structured Assessment of Violence Risk in Youth

**DOI:** 10.3390/healthcare11222952

**Published:** 2023-11-12

**Authors:** Elena Ortega-Campos, Leticia De la Fuente-Sánchez, Flor Zaldívar-Basurto, Mery Estefanía Buestán-Játiva, Juan García-García

**Affiliations:** Health Research Center (CEINSA), University of Almería, 04120 Almería, Spain; elenaortega@ual.es (E.O.-C.); lfuente@ual.es (L.D.l.F.-S.); flor@ual.es (F.Z.-B.); mbj335@ual.es (M.E.B.-J.)

**Keywords:** child-to-parent violence, youth offenders, protective factor, recidivism, risk factor, SAVRY

## Abstract

Child-to-parent violence occurs when children engage in violent behaviour towards family members; the principal victim is often the mother. The risk assessment instruments used to identify the risk and protective factors in youth offenders who perpetrate child-to-parent violence are not specific to this type of offense. This study aims to describe the child-to-parent violence group in relation to the risk and protective factors they present in comparison with the group of young people who committed an assault offence. The sample for this study consists of two groups of youth offenders. The first group committed child-to-parent violence, and the second group has committed a violent crime against individuals to whom they are not related. Young people who commit child-to-parent violence have higher scores on the SAVRY risk factors and lower scores on the SAVRY protective factor than young people who have committed an assault offence. The results reveal the importance of identifying the risk and protective factors presented by youth offenders who commit child-to-parent violence in order to create specific intervention programs for the needs and strengths presented by this group of young people.

## 1. Introduction

Child-to-parent violence (CPV) is a form of intrafamily violence defined as the set of repeated behaviours of physical, psychological, and financial violence carried out by children towards their parents or those performing the parental role [1,2]. Estimates of the worldwide prevalence of CPV span the range of 5–21%. Specifically, in relation to verbal, psychological, and emotional CPV, the incidence rises to 22–93% [3,4]. In Spain last year, a total of 4332 investigations were opened against young people aged 14–18 for violence within the family [5]. Despite the number of investigations opened each year in Spain for this offence, only the most serious cases, representing around 10–15% of the total, are reported [6,7]. The hidden numbers for CPV that do not appear in official statistics are very considerably in excess of the number of formal reports made for this offence [8], being so frequent that other channels of intervention outside the judicial circuit are used.

The family problem of child-to-parent violence has been ignored for many years, due in large part to the silence of the families affected [9]. Parents who are attacked by their children find it difficult to ask for help because they fear reprisals, feel shame or fear, or think they are themselves to blame. The denial or minimisation of the actions of children towards their parents is one of the principal limitations in addressing CPV because it is rendered all the more invisible. Parents are in an ambivalent situation in which they are the victims of abuse perpetrated by their children and, at the same time, they protect their children. This once again means that this form of violence is relegated to the private family domain [8,9].

Violence perpetrated by children towards their parents affects the integration of young people and their families in society, generates negative effects in different areas of life for parents, and increases the likelihood that children will go down a path of offending [10]. Given the prevalence of CPV and the consequences of these behaviours for both parents and their children, it is essential to study the young people who exhibit these behaviours and provide specialist interventions for them [11,12].

The approach to criminal antisocial behaviour focuses on evaluation and intervention in relation to the risk and protective factors presented by a young offender, on the basis of Andrews and Bonta’s [13] Risk, Need, and Responsivity (RNR) Model. Different studies have confirmed that young people who perpetrate CPV have different patterns and dynamics relative to young people who engage in antisocial conduct that constitutes different types of offence [11,14,15,16]. The existence of specific factors associated with CPV has been key to investigations into the criminological profile of CPV offending, which has advanced the prevention of and intervention in CPV [3,17,18,19]. The principal risk factors seen in young people who have engaged in CPV behaviours can be grouped into three blocks: individual, family, and social factors [6,20,21,22].

The most notable factors in the individual block are psychological distress, negative self-concept, low self-esteem, high impulsivity, high aggressivity, an external locus of control, low academic performance, an evasive problem-solving style, a negative attitude towards problems, behavioural problems, substance use, psychopathological or clinical symptoms, and having been a victim of abuse [19,23,24,25,26,27].

Among factors related to the family, those that stand out are a permissive and/or inattentive style of parenting, violence between parents, frequent family conflict, problems associated with parents, and victimisation of the child in conflict [17,18,28,29]. Conversely, in the social factor block, the factors considered significant in CPV are acceptance of violent social attitudes, relationships with peers with behavioural problems who attack their own parents, having been a victim of bullying at school, and inappropriate and abusive use of information technology (social media) [30,31,32,33]. Finally, researchers have considered the protective factors associated with CPV, most notably the existence of prosocial plans for the future, social and family support, open family communication, an indulgent style of parenting (emotional care and loving warmth), and perceived social resources [31,34,35,36].

In studies undertaken to learn about and understand the phenomenon of CPV, it is important to be mindful of the characteristics of the selected sample. Studies may focus on young people drawn from the general population, clinical samples, or young people who have been reported for CPV (as in the case of this work). Another relevant factor is the type of violence perpetrated by young people towards their parents (psychological, physical, financial, or a combination of several) [37,38]. In order to identify and study the group of young people who carry out CPV behaviours, specific instruments have been developed for this group [39], notably: Abusive Behaviour by Children—Indices (ABC-I), Adolescent Domestic Battery Typology, Child-to-Parent Aggression Questionnaire (CPAQ), Child-to-Parent Violence Questionnaire (CPV-Q), Child-to-Parent Violence Risk Assessment Tool (CPVR), and Child-to-Parent Violence Functions Scale (CPV-F) [31,38,40,41,42,43,44].

In the Youth Criminal Justice Service, the use of instruments that predict the risk of re-offending is common in order to determine the risk and protective factors for each young person who has committed a crime involving antisocial behaviour and passes through the criminal justice system. The use of such instruments helps criminal justice staff to plan, implement, and evaluate individualised plans for the path of each young person through the Youth Criminal Justice Service. The most used instruments internationally include the Youth Level of Service/Case Management Inventory (YLS/CMI) [45] and the Structured Assessment of Violence Risk in Youth (SAVRY) [46], which are also used by young people involved in CPV [6,47].

Information gathered through the application of these tools is used to identify individuals at high risk of reoffending, guide legal decisions regarding the intensity of interventions or community reintegration, and help therapists identify targets to reduce the risk of recidivism. The predictive validity of youth risk assessment tools may change, as the individual weights of specific risk and protective factors may vary across young people [48,49]. While there are very few publications available that address developmental differences in risk assessment in the juvenile offender population, Palanques et al. [3] in their study found that the CPV group had mostly committed CPV, whereas the comparison group had tended to commit property crimes. In addition, the results showed that the CPV group had a higher risk profile than the comparison group. The family circumstances, substance abuse, and personality behaviour subscales of the YLS/CMI were able to predict CPV among these youths [50].

These studies have focused primarily on factors that contribute to risk. Aspects that reduce the likelihood of recidivism have received much less attention. Protective factors appear promising for improving risk prediction and management [51]. This view is supported by a growing body of research that increasingly demonstrates the vital role of protective factors in reducing recidivism [51,52]. Following these advances, several youth risk assessment tools have, to some extent, incorporated protective factors. The SAVRY, for example, is one of the most widely used tools and currently the most widely used measure of protective factors in juvenile offenders [53]. These findings demonstrate the need for a detailed assessment of adolescents who have committed CPV offences in order to tailor intervention measures and, broadly speaking, reduce the risk of recidivism [52,54].

In the context we have described and the findings of researchers in the area of CPV, this paper has several objectives. Firstly, to describe the CPV group in relation to the risk and protective factors they present in comparison with the group of young people who committed an assault offence. Secondly, to determine whether the SAVRY [46], an instrument for predicting antisocial behaviour, adequately discriminates between young people who have committed a CPV offence and a group of young people who have committed an assault offence (section 147 et seq. of the Criminal Code). Thirdly, to determine what risk and protective factors the young people in these two groups share and whether the CPV group can be differentiated in terms of risk and protective factors, in order to assess the usefulness of SAVRY for the future planning of specific interventions for each group of young offenders.

## 2. Method

### 2.1. Participants

The sample for this research is made up of a total of 175 young offenders from a juvenile court. All young offenders who committed a CPV or assault offence for one year (January to December) were included in this study. The different sample sizes of the groups (25 for CPV and 150 for assault) are due to the fact that all young people who committed these offences were selected. This was carried out in order to obtain a global and real picture of the phenomenon under study. The data were collected retrospectively from the young people’s judicial files. Specifically, a study year was selected for the commission of the base offence (CPV vs. assault), and thereafter, recidivism was measured for a period of two years after that calendar year. Data were collected three years after the commission of the offence under study in this paper.

### 2.2. Instruments and Variables

#### 2.2.1. Structured Assessment of Violence Risk in Youth

SAVRY [46] is an instrument for the assessment of risk in young people in conflict with the law. It comprises four factors, three related to the risk presented by a young person: history (10 items), social (6 items), and individual (8 items), and one protective factor that has 6 items. SAVRY comprises a total of 24 items related to risk behaviours with three possible responses (low, moderate, and high) and 6 items in the protective factor with two possible answers (present or absent). For each factor (history, social, individual, and protective), a partial score is obtained, and a total score for the level of risk presented by a young person can also be calculated. In this work, the Spanish version of SAVRY [55] has been used.

In order to examine the reliability of scores for the instrument, we estimated Cronbach’s alphas and confidence intervals for the partial and total scores, which gave the following values: α = 0.780, 95% CI (0.728, 0.824) for history; α = 0.683, 95% CI (0.604, 0.749) for social; α = 0.720, 95% CI (0.658, 0.774) for individual; α = 0.825, 95% CI (0.782, 0.860) for the protective factor; and α = 0.879, 95% CI (0.852, 0.902) for the total risk score [56].

#### 2.2.2. Re-Offending and Past Offending

Re-offending was defined as the opening of a further formal investigation into a young person by the public prosecutor. The follow-up period for re-offending was two years from the commission of the criminal antisocial behaviour that led to the inclusion of a young person in this study [57,58,59,60]. Past offending by a young person was measured by the presence or absence of a prior criminal case in their record. The cut-off was two years prior to the commission of the criminal antisocial behaviour that led to the inclusion of a young person in this study.

### 2.3. Procedure

The data-gathering process was conducted in a youth court. The information required to complete the SAVRY was collected retrospectively from the court records of the young offenders. Those files included police information about the incident reported, the formal investigation of the events, a psychological-social-educational report prepared by the youth court’s specialist services, and the sentence imposed by the youth judge. On the basis of the young person’s court file, the information gathering protocol drawn up for the study was completed with sociodemographic variables, information concerning re-offending, the criminal history of each young person, and SAVRY.

Two of the authors acted as data coders. One of the authors coded 100% of the court files, while the other coded 30% of files selected at random. The agreement between coders was above 95%, and discrepancies were resolved by consensus. Both coders have doctorates in psychology, and one of them has more than twenty years’ experience in court and forensic psychology.

The research investigation followed the recommendations of the Risk Assessment Guidelines of the Evaluation of Efficacy (RAGEE) Statement [61] and was approved by the Ethics Committee of the University of Almería (UALBIO2020/017) within the framework of wider research.

### 2.4. Desing

This research has been carried out following the guidelines of an ex post facto design.

### 2.5. Data Analysis

We estimated Cronbach’s alpha reliability for partial and total SAVRY scores in order to assess the internal consistency of the instrument. The reliability coefficients were calculated in accordance with the recommendations of George and Mallery [62].

Descriptive statistics were estimated for sociodemographic variables and partial and total SAVRY scores. In order to determine whether there are differences in scores across the different youth offender profiles studied, non-parametric tests of differences of means were performed (Mann-Whitney’s U). The contrast statistic was accompanied by an estimate of effect size [63,64].

In order to quantify the predictive force of SAVRY to predict re-offending, the area under the curve (AUC) was calculated for scores attained using the instrument. To interpret the AUCs, we took as reference points: AUCs in the range 0.55–0.63 have low predictive value; those in the range 0.64–0.70 have moderate predictive value; and those above 0.71 have good predictive value, complemented by estimation of effect size using Cohen’s index [65,66].

Statistical analysis was carried out using SPSS version 28 (IBM Corp., Armonk, NY, USA) and JASP version 16.4 (University of Amsterdam, Amsterdam, The Netherlands).

## 3. Results

The sample in this study comprises a total of 175 individuals under the age of 18, in respect of whom a procedure had been opened in the youth court for the commission of an offence under Spanish law relating to youth offenders. Under the Youth Criminal Liability Act 2000 [67], offenders aged 14–17 are tried in a youth court.

In terms of sociodemographic data, the young participants in this study are mainly boys (70.3%), of Spanish nationality (80.6%), and have a mean age of 15.68 (1.072). The minority group is those aged 14 at the time of their offence.

In relation to variables associated with the specific offences committed, 85.7% committed an assault, and 14.3% committed an offence considered CPV. For all young people, 83.2% of offences committed have a single victim, 50.6% of victims are under 18 years of age, and 50.9% of victims are males.

For overall variables concerning re-offending, 23.4% of the young people have committed offences in addition to those considered in this study, and 33.7% of the young people re-offend within a two-year follow-up period (Table 1).

In relation to the sex of the youth offenders, in the group that committed offences of assault, 71.3% were males; in the CPV group, the percentage of males falls to 64%. No statistically significant differences were found between the groups in relation to the variable of sex.

With respect to the variable of the age of a young person at the time of commission of criminal antisocial behaviour, there were also no statistically significant differences between the two groups. In the group that committed assault, the greatest percentage of young people are aged 16 (36%) and 17 (29.3%), whereas in the group of young people who committed CPV, the most heavily represented ages are 15 (36%) and 17 (32%). Similarly, there were no statistically significant differences in relation to the nationality of the young offenders (*p* = 0.938); the percentage of Spanish nationals in both groups was approximately 80%.

In relation to the number of victims of criminal antisocial behaviour, no statistically significant differences were found (*p* = 0.363). However, in the Assault group, the majority of offences (84.5%) had one victim, while in the CPV group, 76% of offences had one victim and 24% had two victims.

Statistically significant differences were found both in relation to the sex and the age of the victims as a function of the offence committed (*p* < 0.001). In relation to the sex of the victims, in the group of young people who committed assault, a majority (58.8%) of the victims are male, 32.4% are women, and 8% are of both sexes. Conversely, in the CPV, the victims are mostly (84%) women, of both sexes in 12% of cases, and in only one case was the victim male. In relation to the age of the victims, in the Assault group, 57.8% were minors, compared to 40.1% of adult victims. In distinction, in the CPV, the victims were adults.

In the variables related to the criminal records of the offenders, both past and future, no statistically significant differences were found. Around 23–24% of the young people who committed both offences had past convictions, and in relation to subsequent re-offending, in the Assault group, some 34.7% of the young people re-offended, compared to 28% in the CPV group (Table 2).

**Figure 1:**
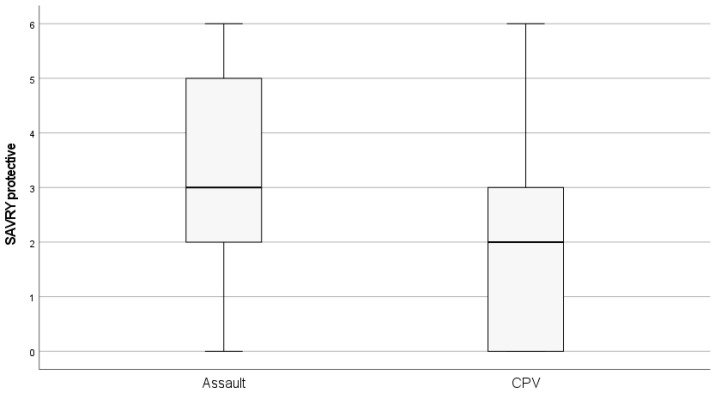
Protective factor scores for CPV and Assault groups.

As can be seen in Table 3, we found statistically significant differences for all the comparisons made between the Assault group and the CPV group. Specifically for the scores for historical risk factors, the Assault group has a mean score of 2.947 (2.877) and the Intrafamily Abuse group has a mean score of 6.040 (3.102), with an effect size of d = 0.794. In the scores for social risk factors, the Assault group has a mean score of 1.660 (2.213), and the CPV group has a mean score of 2.680 (1.952), with an effect size of d = 0.464. In scores for the individual component, the mean score is estimated at 2.427 (2.375), compared to the CPV group, which has a score of 4.680 (2.577) and an effect size of d = 0.627. In the protective factor, the mean score for the Assault group was 3.307 (1.904) and for the CPV group it was 1.800 (1.732) (Figure 1), with an effect size of d = 0.549. Finally, in total SAVRY scores, the Assault group has a mean score of 5.087 (6.821) and the CPV group has a mean score of 11.760 (6.747), with an effect size of d = 0.738. According to the effect sizes found, in the historical risk factor scores, the differences found indicate a large effect between the two groups, while in the other comparisons, the effects found were moderate (Table 3).

In order to further explore the differences found, each of the items that make up the SAVRY is analysed. Table 4 shows the mean scores and standard deviations for each SAV RY item with the *p*-value of the difference of means between groups, the value of the effect size, and the 95% confidence interval. In the historical risk factor, statistically significant differences were found between the assault group and the CPV group for the following items: history of violence (*p* < 0.001, d = 0.873); early initiation of violence (*p* < 0.001, d = 0.379); past supervision/intervention failures (*p* < 0.001, d = 0.531); history of self-harm or suicide attempts (*p* < 0.001, d = 0.178); childhood history of maltreatment (*p* = 0.020, d = 0.166); early caregiver disruption (*p* = 0.033, d = 0.252); and poor school achievement (*p* = 0.011, d = 0.305).

In the social/contextual risk factor, statistically significant differences were found in the poor parental management item (*p* < 0.001, d = 0.792). This was a large difference given the effect size found. In the individual/clinical risk factor, statistically significant differences were found between the Assault group and the CPV group in the following items: negative attitudes (*p* = 0.007, d = 0.311); risk taking/impulsivity (*p* = 0.045, d = 0.262); substance-use difficulties (*p* = 0.017, d = 0.145); anger management problems (*p* < 0.001, d = 0.604); attention deficit/hyperactivity difficulties (*p* < 0.001, d = 0.292); and poor compliance (*p* = 0.018, d = 0.287). A moderate effect was found in the Anger management problems item and small effects for the other items. Finally, in the protective factor, differences were found between the Assault group and the CPV group in the following items: prosocial involvement (*p* = 0.005, d = 0.369); strong social support (*p* = 0.005, d = 0.277); strong attachments and bonds (*p* < 0.001, d = 0.516); and positive attitude towards intervention and authority (*p* = 0.008, d = 0.352). A moderate effect was found in the strong attachments and bonds item, and small effects for the other items.

Finally, the AUCs were estimated for partial and total SAVRY scores. All the estimated curves were statistically significant. Specifically, the following estimates were found for each factor: SAVRY Historical (AUC = 0.805, 95% CI [0.723, 0.888]), SAVRY Social (AUC = 0.687, 95% CI [0.596, 0.779]), SAVRY Individual (AUC = 0.747, 95% CI [0.644, 0.851]), SAVRY Protective (AUC = 0.281, 95% CI [0.174, 0.389]), and SAVRY Risk Total Score (AUC = 0.786, 95% CI [0.696, 0.877]). The historical, individual and protective factors and the Risk Total Score have good predictive power, whilst the social dimension has a moderate predictive power (Table 5).

## 4. Discussion

This work had several research objectives. First, to describe the CPV group in relation to the risk and protective factors they present in comparison with the group of young people who committed an assault offence. Second, to establish whether SAVRY distinguished between young people who had engaged in antisocial behaviour constituting CPV and young people who had committed other types of assaults. The results presented provide evidence of how the groups studied present different scores in all dimensions of SAVRY. The CPV group has higher mean scores than the Assault group in risk factors (historical, social, individual, and Risk Total Score), while in the protection factor, the group that obtains a higher score is the injury group, which suggests that the CPV group will need a more intensive intervention. The estimated effect sizes for the historical factor are large; the effect sizes are moderate for the other factors. In relation to SAVRY’s predictive capacity, moderate and high values were obtained for the risk and protective factors and for the total score, making SAVRY useful for evaluating protective and risk factors differentially with respect to other assaults.

In relation to our third objective concerning the characteristics of the two study groups (CPV vs. Assault), significant differences and moderate to high effect sizes were found for the different SAVRY items. In the CPV group, the presence of factors reported in the specialised literature, such as higher levels of previous violence, is noteworthy [68], poor academic performance [69], deficits in parental management [70], problems with emotional regulation, and impulsivity [12].

Consistent with previous works, it is evident that there are differentiating characteristics or specific factors between young people who commit CPV and young people who commit antisocial behaviours that constitute other offences [15,17,19,71]. As well as implementing specific programmes and interventions, systematic reviews have shown the need for further research into CPV, with special emphasis on what makes interventions with this group of young people effective, what needs and outcomes they address, and the implications of the identification of and targeted work with this group [72].

At the international level, there are various CPV-specific programmes: Step-Up Building Respectful Family Relationships [73], the Break4Change Programme [74], and Responding to Child to Parent Violence [75], which have reported significant improvements, the end of abuse of parents, and lower rates of re-offending. Various programmes are used to address CPV in Spain, with positive results and low re-offending rates [76,77] and high success rates of 93–97.5% [78,79]. In 67.7% of cases, there has been total remission of CPV and improvements to various aspects of family life [78,79,80].

A fundamental aspect of any programme is evaluation, so that its operation can be reviewed and improved [81]. Research into CPV interventions can offer valuable information about how well the programmes followed for CPV by Youth Justice work. Some of the fundamental elements of these programmes have to do with the importance of parental involvement in any intervention, drawing up long-term life plans, relationships with peers who are not problematic, and avoiding substance use [72,82,83,84,85]. We should also mention the comparative lack of information about reviews of the effectiveness of programmes, the difficulty of follow-up, and the importance of the proper assessment and management of risk and protective factors [84,85]. Those factors are important to making the appropriate interventions in CPV and to reducing the risk that a young person will re-offend [12,84,86].

It is essential to assess and manage the risk of re-offending before and after an intervention, with instruments created for that purpose that can help expert staff identify and study variability over the course of a programme or intervention in the risk and protective factors presented by youth offenders.

A number of studies have shown the predictive validity of those instruments to predict the risk of re-offending in young people subject to a criminal sentence; however, there was insufficient data as to their effectiveness in the assessment of personal characteristics associated with specific offences such as those that constitute CPV [87,88,89,90,91]. This work is a step forward in research with young people who have committed CPV in that it provides data about the use in this group of one of the instruments most commonly used internationally to predict the risk of re-offending. SAVRY has shown that it possesses the capacity both to identify CPV young offenders and to make predictions for that group. Expert staff who work in youth justice can use SAVRY with CPV young offenders as part of the process of identifying and planning personalised interventions with these young people.

### Limitations

This study represents an advance in the knowledge of the profiles of young people who have committed a CPV offence by comparing this group with another group of young offenders who have committed an offence of a violent nature but not directed at family members. However, several aspects should be improved in future work. Firstly, the sample size of the CPV group: this type of offence is very specific, and it is therefore relatively difficult to find young people convicted of this offence. However, in future studies, it would be advisable to increase the sample size of the CPV group. One option could be to increase the period of commission for the offence. Another aspect to be taken into account should be not to work only with a generic risk prediction instrument but to complement it with a specific instrument for young people who have committed a CPV offence in order to be able to plan specific interventions for this group.

## 5. Conclusions

Young people who have committed a CPV offence present a specific profile compared to young people with other non-violent offenses. In comparison with other types of violent crimes (e.g., assault), similarities and differences have been found in the risk and protective factors presented by both groups. Knowing the differentiating profile of each group of young offenders helps in the planning of individualised interventions in juvenile justice. The SAVRY instrument, which is not specific for youth with CPV offences, has demonstrated its ability to discriminate between youth with violent assault and CPV offences and its ability to identify individualised profiles. This instrument has proven to be a useful tool in the identification of youth with CPV offences, facilitating the work of juvenile justice technical teams by being able to use the SAVRY for all young offenders, regardless of the offence committed.

## Figures and Tables

**Table 1:** Frequency and percentage of variables in young people.

Variables	% (n)	Variables	% (n)
Sex of minor		Age of minor	
Male	70.3 (123)	14 years	16.6 (29)
Female	29.7 (52)	15 years	28.6 (50)
Nationality		16 years	25.1 (44)
Spanish	80.6 (141)	17 years	29.7 (52)
Other country	19.4 (34)	Number victims	
Offence committed		1	83.2 (144)
Assault	85.7 (150)	2	13.9 (24)
CPV	14.3 (25)	3 o +	2.9 (0.5)
Age victims		Sex victims	
Minors	50.6 (87)	Male	50.9 (88)
Adults	46.5 (80)	Female	39.9 (69)
Both	2.9 (5)	Both	9.2 (16)
Prior offences		Re-offending	
Yes	23.4 (41)	Yes	33.7 (59)
No	76.6 (134)	No	66.3 (116)

**Table 2:** Frequency and percentage of offender variables for the Assault and CPV groups.

	Assault	CPV	Pearson’s Chi-Squared	*p*-Value
Variables	% (n)	% (n)		
Sex of minor				
Male	71.3 (0.107)	64 (0.16)	0.552	0.458
Female	28.7 (0.43)	36 (0.9)		
Age of minor				
14 years	17.3 (0.26)	12 (0.3)	1.289	0.732
15 years	27.3 (0.41)	36 (0.9)		
16 years	36.0 (0.39)	20 (0.5)		
17 years	29.3 (0.44)	32 (0.8)		
Nationality				
Spanish	80.7 (0.121)	80 (0.20)	0.006	0.938
Other country	19.3 (0.29)	20 (0.5)		
Number victims				
1	84.5 (0.125)	76 (0.19)	3.189	0.363
2	12.2 (0.18)	24 (0.6)		
3 o +	3.3 (0.5)	--		
Sex victims				
Male	58.8 (0.87)	4 (0.1)	27.118	<0.001
Female	32.4 (0.48)	84 (0.21)		
Both	8.8 (0.13)	12 (0.3)		
Age victims				
Minors	57.8 (0.85)	--	30.645	<0.001
Adults	40.1 (0.59)	100 (0.25)		
Both	2.0 (0.3)	--		
Prior offences				
Yes	23.3 (0.35)	24 (0.6)	0.005	0.942
No	76.7 (0.115)	76 (0.19)		
Re-offending				
Yes	34.7 (0.52)	28 (0.7)	0.426	0.514
No	65.3 (0.98)	72 (0.18)		

**Table 3:** Descriptive statistics, *p*-value, and estimated effect size for SAVRY scores.

		n	M (SD)	*p*-Value	Cohen’s d
SAVRY_historical_	Assault	150	2.947 (2.877)	<0.001	0.794
	CPV	25	6.040 (3.102)		
SAVRY_social_	Assault	150	1.660 (2.213)	0.002	0.464
	CPV	25	2.680 (1.952)		
SAVRY_individual_	Assault	150	2.427 (2.375)	<0.001	0.627
	CPV	25	4.680 (2.577)		
SAVRY_protective_	Assault	150	3.307 (1.904)	<0.001	0.549
	CPV	25	1.800 (1.732)		
SAVRY_RTS_	Assault	150	5.087 (6.821)	<0.001	0.738
	CPV	25	11.760 (6.747)		

**Table 4:** Means, standard deviations, *p*-value, and Cohen’s d for SAVRY items.

		**M (SD)**	***p*-Value**	**Cohen’s d**
Historical risk				
1. History of violence	Assault	1.233 (0.440)	<0.001	0.873
	CPV	2.080 (0.640)		
2. History of nonviolent offences	Assault	1.133 (0.378)	0.985	0.002
	CPV	1.120 (0.332)		
3. Early initiation of violence	Assault	1.100 (0.323)	<0.001	0.379
	CPV	1.440 (0.583)		
4. Past supervision/intervention failures	Assault	1.127 (0.389)	<0.001	0.531
	CPV	1.720 (0.792)		
5. History of self-harm or suicide attempts	Assault	1.020 (0.182)	<0.001	0.178
	CPV	1.240 (0.597)		
6. Exposure to violence in the home	Assault	1.207 (0.559)	0.154	0.134
	CPV	1.400 (0.764)		
7. Childhood history of maltreatment	Assault	1.100 (0.414)	0.020	0.166
	CPV	1.280 (0.614)		
8. Parental/caregiver criminality	Assault	1.207 (0.571)	0.730	0.031
	CPV	1.200 (0.500)		
9. Early caregiver disruption	Assault	1.333 (0.642)	0.033	0.252
	CPV	1.640 (0.810)		
10. Poor school achievement	Assault	2.487 (0.809)	0.011	0.305
	CPV	2.920 (0.277)		
Social/contextual risk				
11. Peer delinquency	Assault	1.467 (0.766)	0.266	0.139
	CPV	1.600 (0.764)		
12. Peer rejection	Assault	1.193 (0.487)	0.356	0.085
	CPV	1.120 (0.440)		
13. Stress and poor coping	Assault	1.220 (0.447)	0.438	0.084
	CPV	1.280 (0.458)		
14. Poor parental management	Assault	1.360 (0.616)	<0.001	0.792
	CPV	2.160 (0.624)		
15. Lack of personal/social support	Assault	1.233 (0.607)	0.260	0.107
	CPV	1.320 (0.627)		
16. Community disorganisation	Assault	1.187 (0.483)	0.798	0.024
	CPV	1.200 (0.577)		
Individual/clinical risk				
17. Negative attitudes	Assault	1.240 (0.487)	0.007	0.311
	CPV	1.480 (0.510)		
18. Risk-taking/impulsivity	Assault	1.393 (0.554)	0.045	0.262
	CPV	1.720 (0.792)		
19. Substance-use difficulties	Assault	1.060 (0.312)	0.017	0.145
	CPV	1.240 (0.597)		
20. Anger management problems	Assault	1.140 (0.367)	<0.001	0.604
	CPV	1.720 (0.678)		
21. Low empathy/remorse	Assault	1.033 (0.180)	0.056	0.105
	CPV	1.120 (0.332)		
22. Attention deficit/hyperactivity difficulties	Assault	1.093 (0.335)	<0.001	0.292
	CPV	1.360 (0.569)		
23. Poor compliance	Assault	1.320 (0.594)	0.018	0.287
	CPV	1.640 (0.757)		
24. Low interest/commitment to school	Assault	2.147 (0.915)	0.235	0.164
	CPV	2.400 (0.764)		
Protective factors				
1. Prosocial involvement	Assault	0.500 (0.502)	0.005	0.369
	CPV	0.200 (0.408)		
2. Strong social support	Assault	0.867 (0.341)	0.005	0.277
	CPV	0.640 (0.490)		
3. Strong attachments and bonds	Assault	0.813 (0.391)	<0.001	0.516
	CPV	0.400 (0.500)		
4. Positive attitude towards intervention and authority	Assault	0.567 (0.497)	0.008	0.352
	CPV	0.280 (0.458)		
5. Strong commitment to school	Assault	0.400 (0.492)	0.056	0.244
	CPV	0.200 (0.408)		
6. Resilient personality traits	Assault	0.160 (0.368)	0.301	0.097
	CPV	0.080 (0.277)		

Assault (n = 150); CPV (n = 25).

**Table 5:** AUCs, 95% CI, and effect size for SAVRY dimensions.

	Area	*p*-Value	CI 95%	Cohen’s d
SAVRY_Historical_	0.805	<1.001	[0.723, 0.888]	1.216
SAVRY_Social_	0.687	1.003	[0.596, 0.779]	0.689
SAVRY_Individual_	0.747	<0.001	[0.644, 0.851]	0.940
SAVRY_Protective_	0.281	<0.001	[0.174, 0.389]	0.820
SAVRY_Risk Total Score_	0.786	<0.001	[0.696, 0.877]	1.121

## Data Availability

The data presented in this study are available on request from the corresponding author.
